# School Diseases—Preventable, but Not Prevented

**Published:** 1906-09-15

**Authors:** 


### The Hospital

A Journal of

The Medical Sciences and Hospital Administration.

Vol. XL.—No. 1,043. SATURDAY, SEPTEMBER 15, 1906.

### SCHOOL DISEASES—PREVENTABLE, BUT NOT PREVENTED.

The re-opening of the schools after the holiday recess directs attention to the means at present adopted to ensure cleanliness for the reception of the scholars. A clean school means healthy scholars, and the education authorities have recently instituted what they consider an effective method of keeping them healthy by the appointment of medical inspectors. This method does not go far in preventing the spread of school diseases. A pupil with ringworm may have infected the floors of a school-room long before medical inspection has revealed the condition. By the removal of the child, modern school hygiene apparently has done all that it thinks necessary. Nothing is done to destroy the germs of the disease, which have been blown about and have been allowed to settle on the school-room floor along with the scurf from the child's head. If one thing can be demonstrated more clearly than another it is that cultures of every zymotic disease to which children are liable can be obtained from the dust on the school-room floors. There they can be found in an undiluted and virulent condition ready to infect every susceptible child. That this occurs there can be no doubt. It is no small matter to have something like 3,754 cases of scarlet fever occurring among children of school age in the London district in one year—a figure which brings London into very unfavourable comparison with Paris, Brussels, and some other European capitals. The great bulk of diphtheria cases, as well as the mortality rate, occurs among younger children, and calls urgently for care of the infant school-rooms. Thus the number of children affected in London at five years of age amounted in one year to 2,316, the fatal cases numbering 186; between five and ten years, 747, with 26 deaths; between ten and fifteen years 321, with 6 fatalities. No large city in Europe or America has anything approaching this grave condition of affairs. London compares worse than any European capital and very much worse than New York. As regards measles and whooping-cough, which are intensely infectious diseases at an early stage, by the time 1 per cent. of children attending is infected, the infection, we may be sure, has spread through the whole school, and all those susceptible will have the disease whether the schools are closed or not. These two infectious diseases cause more deaths than all the others put together. School hygiene in this country has been hitherto almost completely neglected, whereas in European and American capitals its importance has been understood and practised for many years. These cities are now reaping the benefit of the adoption of scientific methods, while London is still groping about like the man with the muckrake, looking for disease germs in the air instead of destroying them in the dust of the school floors. This condition of helplessness in the presence of filth is demonstrated in a Blue Book which has recently been issued by the Government on the ventilation of the House of Commons, which clearly proves that even in the chamber of wisdom bacilli present themselves in dangerous colonies undismayed under the very lips and noses of our august and learned legislators. What is true of the House of Legislature is ten thousand times more true of schools and institutions in which children are crowded together by thousands. A school investigation is about to take place to ascertain whether any of the school-children, and if so how many, are suffering from consumption. The result is hardly in doubt, considering how rampant the malady is in every portion of the London district, but especially so in the crowded districts. Stepney, for example, takes the lead with 648 deaths from consumption in 1904. Islington comes next with 532, Southwark follows with 501 fatal cases of consumption, and Lambeth yields 448 emaciated corpses from the same malady. The number actually ill must be appalling, and necessarily a source of serious danger whenever, as in schools, the children are crowded thickly together. The Education health officer, Dr. Kerr, will deserve thanks if he succeeds in teaching the children so affected how to disinfect sputum; but still more if he can impress on the teachers and the authorities the pre-eminent duty of neutralising the mischief by the nightly application to the tuberculous floors of some effective sterilising agent. The great bulk of school diseases are entirely preventable if only the principles of thorough disinfection, as they are known to present-day science, are properly and strenuously applied. The same principles with all stringency and regulation which apply to the surgical department of a hospital should be made to apply with equal stringency to a school-room. School fevers are precisely of a similar character as what used to be known as "Hospitalism," and school infection and the deaths per annum among children of school age are due to a similar cause. They are to be removed by applying the same remedy to the school and institution that was and is now applied in the surgical ward of a hospital. This "Schoolism," carrying off as it does so many of our fairest and brightest in the early days of childhood and youth, is a standing shame and signal disgrace to our educational authorities and their systems of hygiene.

